# Development of Coronavirus Treatments Using Neutralizing Antibodies

**DOI:** 10.3390/microorganisms9010165

**Published:** 2021-01-13

**Authors:** Saman Fouladirad, Horacio Bach

**Affiliations:** 1 Department of Medicine, University of British Columbia, Vancouver, BC V6T 1Z, Canada; saman.fouladirad@alumni.ubc.ca; 2 Division of Infectious Diseases, University of British Columbia, Vancouver, BC V6T 1Z, Canada

**Keywords:** coronavirus, SARS-CoV-2, MERS-CoV, SARS-CoV, antibody treatment, neutralizing antibodies

## Abstract

The Coronavirus disease 2019 (COVID-19), caused by the novel coronavirus SARS-CoV-2, was first reported in December 2019 in Wuhan, Hubei province, China. This virus has led to 61.8 million cases worldwide being reported as of December 1st, 2020. Currently, there are no definite approved therapies endorsed by the World Health Organization for COVID-19, focusing only on supportive care. Treatment centers around symptom management, including oxygen therapy or invasive mechanical ventilation. Immunotherapy has the potential to play a role in the treatment of SARS-CoV-2. Monoclonal antibodies (mAbs), in particular, is a relatively new approach in the world of infectious diseases and has the benefit of overcoming challenges with serum therapy and intravenous immunoglobulins preparations. Here, we reviewed the articles published in PubMed with the purpose of summarizing the currently available evidence for the use of neutralizing antibodies as a potential treatment for coronaviruses. Studies reporting in vivo results were summarized and analyzed. Despite promising data from some studies, none of them progressed to clinical trials. It is expected that neutralizing antibodies might offer an alternative for COVID-19 treatment. Thus, there is a need for randomized trials to understand the potential use of this treatment.

## 1. Introduction

The Coronavirus disease 2019 (COVID-19), caused by the novel coronavirus SARS-CoV-2, was first reported in December 2019 in Wuhan, Hubei province, China and since then has caused a worldwide pandemic, with over 61.8 million cases being reported as of December 1st, 2020 [1,2]. 

The coronaviruses are a diverse and large group of viruses that contain a single strand RNA that can be isolated from many different animals and has the potential to spread to humans causing symptoms that range from the common cold to multiorgan failure and ultimately death [3]. Just like the previously identified and highly pathogenic SARS-CoV and the Middle East respiratory syndrome (MERS)-CoV, SARS-CoV-2 is also believed to be a zoonotic virus, using bats as a natural reservoir. However, unlike MERS-CoV and SARS-CoV-2, where the virus moves from bats to a mammalian host, Himalayan palm civet for SARS-CoV, and the dromedary camel for MERS-CoV, the transmission pattern and dynamics of the fast-spreading infection of SARS-CoV-2 is still yet to be determined [4].

As it currently stands, there are no definite approved therapies endorsed by the World Health Organization (WHO) for COVID-19, only supportive care [5]. Treatment centers around symptom management, including oxygen therapy or invasive mechanical ventilation (IMV) in severe cases [6]. Antivirals such as remdesivir are only moderately recommended by the American National Institute of Health (NIH) for severe cases, while lopinavir/ritonavir are not recommended given unfavorable pharmacodynamics and lack of clinical efficacy [6]. Additionally, the use of corticosteroids to reduce cytokine-related pulmonary damage in patients with regard to COVID-19 remains controversial [7], while studies investigating the potential of chloroquine and hydroxychloroquine show mixed outcomes [8].

Immunotherapy has the potential to play a role in the treatment of SARS-CoV-2. Monoclonal antibodies (mAbs), in particular, is a relatively new approach in the world of infectious diseases and has the benefit of overcoming challenges with serum therapy and intravenous immunoglobulins preparations (convalescent plasma) [9], including purity, low risk of blood-borne pathogen contamination, and safety [3]. The ability of mAbs to mimic or bind to specific substrates of interest in the body, blocking or enhancing precise mechanisms to provide therapeutic intervention, has been evaluated for use in treating various diseases.

With the lack of current treatments for COVID-19 and the increase in recognition for the use of mAbs as a promising drug class in infectious diseases, we here reviewed past lessons learned from the different mAbs used in previous studies to treat coronaviruses such as SARS-CoV-1, and MERS to help better equip us with the knowledge to design potential therapeutic target treatments for COVID-19 [10,11,12].

## 2. Methods

Using the PubMed database, relevant articles were identified using Severe Acute Respiratory Syndrome/virology, SARS-CoV-1 antibody, Middle East Respiratory Syndrome Drugs, and MERS-COV antibody, coronavirus antibody therapy, and SARS/MERS neutralizing antibodies. From the resulting articles, studies were selected that identified and tested a novel neutralizing mAb in vitro/in vivo against SARS-CoV-1/MERS-CoV or contributed to the primary research by providing more information about the mechanism of action, neutralizing profile, or epitope mapping of the previously identified antibody. With these inclusion criteria, 30 articles were identified: 12 about SARS-CoV-1, 16 regarding MERS-CoV, and 2 overlapping both. 

## 3. Severe Acute Respiratory Syndrome (SARS)

The Severe Acute Respiratory Syndrome (SARS), caused by a coronavirus, emerged as a life-threatening respiratory infection that led to the 2002–2003 epidemic, and re-emergence in 2003/2004 of 8000 infections and a fatality rate of approximately 10% across 29 countries [13,14,15]. 

The SARS-CoV is a zoonotic virus that is believed to have originated in Chinese horseshoe bats that eventually amplified in live market animals, including palm civets and raccoon dogs, before being transmitted to humans [16,17]. Infected patients have been documented to show signs of atypical pneumonia and severe lung damage, with around 20% developing acute respiratory syndrome, which can be attributed to infection of pneumocytes, epithelial lining, and alveolus of the lung [13,18,19,20,21]. While the primary pathological focus was in the lungs, systemic distribution of infection was frequently observed [21,22]. 

The spike (S) protein of SARS-CoV, a type I transmembrane glycoprotein of around 1255 residues, is essential for binding to human host receptor angiotensin-converting enzyme 2 (ACE2) and mediating the entry of viral components into the cell [23,24,25]. This protein contains two domains, S1 and S2, with S1 having the receptor-binding domain (RBD), a region of 193 residues involved in binding to the receptor ACE2, involved in the internalization of the virus into the host cell. In contrast, S2 contains the two conserved heptad regions (HR1 and HR2) and the fusion peptide necessary for viral–host cell fusion [23,26,27]. These S protein components are thus a target for developing the various treatment strategies to inhibit and neutralize the virus.

### 3.1. IgG and Antibody Variants

#### 3.1.1. CR3014 and CR3022

CR3014 and CR3022 are both IgGs that bind the S protein to prevent its attachment and binding to its cellular receptor ACE2 [28,29,30,31,32]. Specifically, both CR3014 and CR3022 act on the S1 domain within the RBD residues 318 to 510 to block the association of the SARS-CoV with ACE2 to neutralize its activity [33]. 

To test the efficiency of immunoprophylaxis with human mAb, CR3014 was generated and tested for in vivo potency in ferrets. These animals are a reliable model for infection with SARS-CoV to produce a high number of viral replicates with lung lesions [34]. Two sets of experiments were performed. Firstly, a comparison of the CR3014 to control antibody, which showed lower levels of SARS-CoV in lung homogenates, decreased lung lesions, and less shedding of the virus in the throats of ferrets. In the second experiment, the CR3022 was studied for its synergistic activity with CR3014 and breadth activity in protection against SARS-CoV strain HKU-39849 as prevention of neutralization escape of the virus [35]. SARS-CoV variants, with a single mutation of proline to leucine at position 462 (P462L) in the spike glycoprotein region, were generated for neutralization escape from CR3014. CR3022 was identified to neutralize escape variants when exposed with CR3014 by binding non-competitively to the RBD of spike glycoprotein. This non-competitive binding allowed for further synergistic activity when a mix of both CR3014 and CR3022 showed how each of the antibodies target different epitopes of the RBD of the virus to exert their effect. CR3022 was also able to bind to the RBDs of SARS-CoV isolates from civet cats and human isolates with mutations of asparagine to serine at amino acid 479 (N479S), which were only partially covered by CR3014. This synergistic activity also led to less required doses of the antibodies needed to achieve neutralization. 

Given concerns of antibody-dependent enhancement (ADE) of viral replication in macrophages of coronavirus lines, the study aimed to see whether this phenomenon would be exhibited in SARS-CoV with a sub-neutralizing concentration of CR3014/CR3022. Results showed that while macrophages take up the virus, this does not lead to a transition of an abortive to productive virus infection, suggesting that it is less likely to observe the antibody-dependent enhancement (ADE) in vivo [35].

While a synergetic activity against SARS-CoV isolate was observed, it is important to note that an effective immunotherapy against a SARS outbreak would need to cover a wide range of viral isolates that may emerge, as indicated in surveillance data of known animal reservoirs of SARS-CoV that confirmed that the viruses in 2003/2004 were different from the ones responsible for the outbreak in 2002/2003 [35,36]. Therefore, while a combination of CR3014 and CR3022 offers an expanded breadth of activity, early and rapid genotyping of the target neutralizing epitope needs to be conducted to better understand the susceptibly to antibody prophylaxis [37]. Additionally, the CR3014 escape variant generated in vitro under the HKU-39849 strain is a variant that has not been reported in any SARS-CoV viruses. Therefore, the neutralization of this escape variant may not accurately reflect an in vivo case, thus requiring further testing of the CR3014/CR3022 in animal models.

#### 3.1.2. F26G19 and F26G20

In selecting an immunization method to develop or discover mAbs, the response will take a more inherent course if the native virions in their closest form to their natural state could be used as an immunogen. In other words, the selection of antibodies using either an epitope of recombinant proteins versus a whole virus could potentially change the treatment strategies [37,38]. For example, a soluble recombinant monomeric S protein might not be the ideal immunogen to elicit the expected mAb response compared to the natural conformation of the protein [37]. A new approach was used to generated a panel of mAbs against the whole inactivated SARS-CoV [37]. The screening result identified the two potent antibodies F26G18 and F26G19, which exerted their activity by binding within the RBD of S1. Further analysis showed that the antibodies specifically targeted the residues 460-476, blocking the association of RBD of S1 subunit with the ACE2 cellular receptor as demonstrated ex vivo [37]. This finding reinforced the importance of the 33-residue segment within the RBD as an antigen target bound by other neutralizing antibodies, such as m396 and 80R. 

To assess the potential use for treatment, 18H18L (F26G18) and 19H19L (F26G19) chimeric mouse–human mAbs were created. 18H18L was able to bind recombinant spike protein with the same affinity and neutralize the Tor2 strain of SARS-CoV just as well as its parental mAb. Simultaneously, the 19H19L failed to function as a chimera, possibly due to mutational changes in the mAb that made it incompatible with the human Fc regions during chimerization. However, mutational changes in the highly prone S1 region may produce inconsistent humoral responses, thus requiring us to monitor changes continually and study other potential antigenic sites for the target. 

#### 3.1.3. S109.8, S227.14, and S230.15

Whereas most of the past studies had generated monoclonal antibodies against late phase SARS-CoV strains and examined their activity in young animal models, the identification of monoclonal antibodies with neutralization activity at different phases of the disease was pursued [39]. 

Spike glycoprotein variants, representing the zoonotic isolates HC/SZ/61/03 (palm civet) and A031G (raccoon dog); the early phase of GZ02, middle phase CUHK-W1, and late phase Urbani (human), were generated [40]. Then, a large panel of antibodies was tested against these isolates. Results showed that the three most broadly neutralizing monoclonal antibodies S109.8, S227.14, and S230.15, exhibited cross-neutralization across all the spike variants in vitro. The assessment of S230.15 activity against the viral strains GD03-S and SZ16-K479N, were assessed both in vitro and in vivo [39]. Results of this study confirmed the antibody’s neutralization activity across different phases of the epidemic and the binding epitope of S230.15 was mapped. Interestingly, S230.15 bound an epitope in the RBD that overlapped with those of 80R and m396 through the residue T487 [41], preventing the virus from attaching to the ACE2. 

Understanding that certain limitations in the past in vivo studies were observed, lethal homologous and heterologous SARS-CoV were designed to challenge young and aged murine models [40]. These models represented a broad demographic as old age is a strong indicator of poor prognosis and increased respiratory problems in SARS-CoV patients. Separate experiments examined the effects of single antibodies or a cocktail of the three antibodies (S109.8, S227.14, and S230.15) in the presence of various viral strains to investigate differences in viral replications in lungs, weight loss, and other clinical signs between 12-month and 10-week-old mice. 

The results showed that the three antibodies, S109.8, S227.14, and S230.15, were able to reduce the viral load in the lungs of mice when challenged with the early or late phase of human isolates. However, animals challenged with the palm civet virus HC/SZ/61/03 strains were less protected, whereas S109.8 was generally ineffective in protecting the various lethal strains, needing high doses to take effect. In general, aged animals were less protected against deadly strains and less effective at clearing the virus, possibly due to differences in innate immunity across age or difficulty in the transition of IgG molecules traversing epithelial barriers to reach the lung due to age [42,43]. Furthermore, a significant limitation of these antibodies was their narrow window of prophylactic activity, as the most effective antibody (S230.15) showed that it could only protect against clinical disease and neutralize viral activity when administered 1 day before being challenged but failed to protect against clinical disease when administered 1-, 2-, or 3-days post-challenge. Even though the viral levels were reduced with the antibody post-challenge, their ineffectiveness against clinical disease leads them to death, similar to human SARS-CoV cases where clinical conditions have shown to be determinant of patient outcome even with undetectable viral levels [44].

Posing the challenge of adhering to isolates across multiple phases, differences in activity across age, and a very narrow window of prophylactic activity, it is perhaps more feasible to achieve therapeutic results using a cocktail of the three antibodies (S109.8, S227.14, and S230.15). Not only did this mix of antibodies show effectiveness against strains (HC/SZ/61/03) that were not previously protected against using single antibody treatment, but a combination of the antibodies can account for the neutralization of escape mutants that would otherwise be generated by individual antibody treatments. 

#### 3.1.4. 201, 68 and 4D4

As mentioned above, the S1 domain contains the RBD for attachment of the virus to the host ACE-2 receptor. Realizing the importance of the RBD within the S glycoprotein for the fusion of SARS-CoV with its host-receptor, investigations were performed to identify neutralizing antibodies that target this specific region [33,45]. Thus, an ectodomain (amino acid range 1–1190) of the SARS-CoV S glycoproteins from Urbani strains were designed [46]. These constructs were transfected into HuMAb mice (Medarex), known for being transgenic for human immunoglobin genes [46]. The antibodies 201 and 68 showed the highest neutralization activity binding the RBD at residues positioned in the amino acid sequences 490–590 and 130–150, respectively [24,47,48]. 

To examine their immunoprophylaxis in vivo, murine models were given the antibodies 1 day before inoculation of the animals with SARS-CoV Urbani strain. Both antibodies protected the animals as indicated by a significant viral reduction in lungs and turbinate tissue (upper respiratory tract) in a dose-dependent fashion. However, the investigators noted that even with the promising results obtained, SARS treatment with these antibodies required further additional testing with other animal models [46]. Clinical trials were also noted to be planned for antibody 201; however, this was never followed up. 

In follow-up experiments, transgenic XenoMouse, instead of HuMAB, was used to produce human antibodies against SARS-COV. This model is known to generate IgG2 isotypes, which has the advantage of not activating the complement system and having low affinity to the Fc receptor on macrophages, making it less likely to cause ADE as seen in CoV feline infectious peritonitis virus [49,50,51,52]. Analysis performed on XenoMouse immunized with the mentioned ectodomain generated 19 neutralizing antibodies, 18 of which inhibited the RBD binding (at position 318–510 within S1 domain) of the virus to inhibit its attachment to the ACE-2 receptor of the host. In contrast, the antibody 4D4 inhibited the post-binding process. However, the generated antibodies failed to bind the RBD region that was mutated in the viral isolates Sin845, GD01, and GZ0402, except for 4D4, which bound the RBD but with less affinity. Analysis of the binding of the 4D4 antibody was determined to be at the N-terminal of RBD and exerts its effect by inhibiting a post-binding process needed for viral entry. Realizing the high potency of the S1 domain to mutations, which allows the virus to evade neutralization, the effect of non-S1 binding antibodies to recombinant S2 ectodomain, comprising of HR1 and HR2 regions, were tested in the human embryonal kidney 293FT cells. Results showed a generally favorable binding of mAb and inhibiting of entry with pseudoviral variations tested.

Taken together, the results suggest that the S2 domain is an effective target for antibody treatment. This may be due to highly conserved neutralization epitopes of HR1 and HR2 within the S2 domain across clinical isolates. In contrast, the RBD S1 is known to be susceptible to various mutations, rendering the antibody ineffective. Although this statement was promising, changes in the genotype of future evolving viral strains within the S2 domain might not reproduce the results observed. However, it is acknowledged that a cocktail of antibodies targeting different regions of the S protein is more likely to offer better protection across various isolates, as observed when 4D4 combined with the antibodies 1F8 (binds HR1) or 5E9 (binds HR2) showed neutralization of the mutants Sin845, GZ-C and GZ0402 to a greater extent compared to single antibody treatment [53].

#### 3.1.5. 1A9

Studies show evidence of the effectiveness of peptides derived from the HR1 and HR2 regions of infusion glycoproteins for neutralization of retroviruses, paramyxoviruses, and coronaviruses, treatment options using antibodies targeting these conserved domains may serve as an attractive option [54,55,56,57,58].

A study aimed to identify specific antibodies targeting the HR2 domain was conducted using S protein fragments of SARS-CoV, which were derived from *Escherichia coli*. These fragments were used to generate a panel of murine monoclonal antibodies targeting the region comprising residues 1029 to 1192. The resulting 15 antibodies were classified into four different types (Type I, II, III, and IV) based on the antigenic site of activity and the ability to neutralize virus ex vivo at neutralization titers that were relatively lower in comparison to antibodies targeting the RBD of the SARS S glycoprotein [58,59].

In a follow-up study, the properties of the antibody 1A9, belonging to the Type II group, were examined. This antibody was the most effective at blocking viral attachment to cell membrane out of the 15 antibodies generated previously. The cross-neutralization ability of IA9 was examined by testing its ability to prevent the viral entry of SARS-CoV strains of civets and SARS-like CoVs (SL-CoVs). S-pseudotyped virus particles (S-PPS) were generated to carry the S protein of human SARS-COV, civet SARS-CoV (SZ3), and bat SL-COVs (Rp3 and Rf1) strains. These constructs were used to infect the Chinese hamster ovary cell line expressing the ACE2 receptor in the absence or presence of different concentrations of the antibody 1A9. Results showed that 1A9 was able to cross-neutralize across the various strains by binding to a novel epitope located in a loop region between HR1 and HR2 at residues 1111-1130 of the S protein [60] with limitations. For example, analysis of S mutations in escape mutants showed the importance of the aspartic acid at position 1128. A single mutation at D1128A rendered the antibody ineffective in its neutralizing activity, possibly due to some steric hindrance induced in preventing the association of H1 and H2. 

Furthermore, in the design of the strains for the experiment, the RBDs (region 322-496) of the S protein of civet SARS-CoV SZ3, bat SL-CoV Rp3, and Rf1, were replaced with RBD of human SARS-CoV HKU39849 to allow viral attachment of the RBD to human ACE2 receptor. Additionally, the IC_50_ of 1A9 was within 25 to 50 μg/mL, a range much greater in comparison to antibodies that targeted RBDs in human and civet SARS-CoV strains [36,39,61]. This disparity may be because 1A9 had the least effective neutralizing property from the 15 antibodies generated in the early study [36]. Therefore, future studies should assess the rest of the antibodies from the panel to compare ex vivo and in vivo activities. 

### 3.2. ScFv Antibodies

#### 80R

The scFv antibody 80R was developed against the S1domain of SARS-CoV spike protein using a non-immune human antibody library [62]. The interaction of the spike protein with the host ACE2 receptor was inhibited after exposure to the 80R scFv. Specifically, mapping the epitope recognized by 80R showed regions within the N-terminal 261–672 amino acid of the spike protein in the S1 domain of SARS-CoV as critical for blocking of the RBD–ACE2 interaction and preventing syncytium formation in the human embryonic kidney Hek 293T cells. The initial experiment involved testing the 80R neutralization in an assay of susceptible monkey epithelial Vero E6 cells using SARS-CoV units prepared from the Urbani strain [36]. Based on the potent neutralization effect of 80R scFv, a new version of 80R IgG1 was constructed. The follow-up experiment sought to examine activity levels of 80R IgG1 as prophylaxis in vivo using a mouse model [62]. Passive administration of 80R IgG1 was given to mice intraperitoneally one day before being challenged with SARS-CoV (Urbani strain) intranasally. The viral titers in lung tissues were then measured to see the effect of 80R IgG1 versus equivalent amounts of human IgG1. Results showed a greater than four-log reduction in viral load of mice administered 80R IgG1 at doses therapeutically achievable in humans through the binding of 80R to the core region of the S protein via the conformational dependent region of residues 324 to 503 that involved all six-antibody complementarity-determining region (CDR) loops that extend into the concave-like receptor binding surface of S1 [24,37,63].

While the results suggested that 80R IgG1 has the potential to be used in emergency prophylaxis, it is important to consider the effectiveness of such antibodies across multiple strains of SAR-CoV, given the high variance exhibited with amino acid mutational changes in the S protein [64]. For example, this factor of high variance in the strains was examined to assess the effectiveness of 80R IgG1 against civet cats and strains across the 2002/2003 and 2003/2004 outbreaks [65]. Surprisingly, the binding of the 80R IgG1 showed a significant reduction in representative isolates from the first outbreak (Tor2) through mutations with Alaine variants. In contrast, multiple substitutions of amino acids in isolates representing the 2003/2004 outbreak (GD03T) showed a complete loss of binding to 80R. Furthermore, while neutralization activity against representative isolates from the first outbreak (Tor2) and palm civets (SZ3) was still susceptible to 80R IgG1 neutralization, as examined through a pseudovirus system; the GD03T strain showed near-complete resistance. 

Even though 80R IgG1 shows some promising results, many significant limitations were of concerns such as the highly sensitive conformational dependent epitope region, a substantial reduction in binding the ACE2 receptor upon specific amino acid mutations changes, and a lack of neutralization activity against strains from newer generations of SARS-CoV (GD03T). This can be of great concern given the continuance variation of SARS-CoV genomes in animal reservoirs given the high mutational rates. Therefore, appropriate profiling of susceptibly and resistant patterns through rapid genotyping of the S1 neutralization epitope region of 80R in new strains of the virus is important in developing proper prophylaxis management. 

### 3.3. Fab Antibodies

#### m396 and S230.15

Upon acknowledging new viral strains following the second SARS-CoV outbreak in 2003/2004, such as the GD03 that exhibited resistance to neutralization to previous human antibodies, new screenings were performed. The human mAbs m396 and S230.15 were identified as potential targeting and neutralizing antibodies against strains from the first and second SARS-CoV outbreaks [39].

Both m396 and S230.15 were identified following their complex formation with the RBD of the SARS-CoV glycoprotein from a human antibody Fab library consisting of over 10^10^ antibodies [39]. The mechanism of this neutralization is thus attributed to the antibody interfering with the binding of the SARS-CoV RBD with the ACE2 host receptor. Specifically, the highly conserved residues Ile-489 and Tyr-491 of the spike protein were identified as playing an important role for binding at m396′s cleft formed by the CDR loops H1, H2, H3, and L3 [37,39,64]. S230.15 exerts its activity by binding to epitopes on B domains of SARS-CoV to prevent its binding to ACE2 [66].

In vitro analysis was conducted to examine the effect of m396 neutralization of pseudotype viruses with the S glycoprotein representing strains from the first outbreak (Urbani and Tor2 strains) in comparison to strains from the second outbreak (GD03) [39]. The use of m396 achieved neutralization across the different pseudotype strains and in the live SARS-CoV isolate (HKU39849) in Vero E6 cells, whereas the use of S230.15 reached neutralization from palm civet isolates SZ3 and SZ16. When comparing neutralization of recombinant replication-competent SARS-CoV isolates Urbani, GD03, and SZ16, IC_50_ values of 0.6 μg/mL and 1.1 μg/mL were measured for m396 and 80R, respectively, under the same experimental conditions [36]. To compare the correlation of in vitro with in vivo activity, 8-week-old female mice models were administered with m396, S230.15, or control antibodies 24 h before being challenged with recombinant SARS-CoVs (Urbani, GD03, and SZ16). Neutralization was noted to be achieved across the different strains in a dose-dependent fashion, as reflected by lower virus titers in mouse lungs with higher serum-neutralizing antibodies. 

Taken together, the results suggest that m396 and S230.15 can achieve viral neutralization in SARS-CoV isolates across 2002/2003, 2003/2004 outbreaks, and palm civet isolates (SZ3 and SZ16) given their consistent in vitro and in vivo effects. However, it is important to note that while the specific crystal structure of m396 is believed to bind across all variants from the first two outbreaks (and possibly future isolates), it cannot guarantee that new isolates will be neutralized, especially given the variance in the structure of the spike protein within ACE2 binding site [66]. Additionally, while experimental data showed promising results, there is a lack of clinical trials conducted with m396 and S230.15 to determine their clinical efficacy. 

## 4. Middle East Respiratory Syndrome (MERS)

The Middle East respiratory syndrome (MERS), caused by the MERS coronavirus (MERS-CoV), is a novel beta-coronavirus related to the bat coronavirus and the sixth coronavirus to cause human infections [67,68,69]. The virus was first identified in June 2012 in Saudi Arabia and presents very similarly to SARS, often causing respiratory distress and pneumonia. While most of the cases have been in Middle Eastern countries, globally, the MERS-CoV has caused around 2494 human infections (858 deaths) in 27 countries around the world as of November 2019 [70,71]. 

The MERS-CoV genome consists of a single-stranded RNA comprised of 10 open reading frames, in which some are responsible for encoding important viral structural proteins such as including spike (S), envelope (E), membrane (M), and nucleocapsid (N) [72]. As in SARS-CoV, the S protein is the main structure responsible for binding to the host receptor, allowing for viral entry, and thus, is a potential target of many therapeutic drugs. The S protein is comprised of an RBD spanning between the residues 367-588, which is involved in the binding to the extracellular domain of the host receptor dipeptidyl peptidase 4 (DPP4/CD26). While the RBS of SARS-CoV and MERS-CoV share many similarities in their structural domains, their variations in the composition of their receptor binding motif leads them to the binding of different receptors [73]. As with the S2 subunit of other coronaviruses, the MERS-CoV S2 is responsible for membrane fusion via the conformational interplay of the HR1 and HR2 regions. Thus, upon membrane fusion, S2 separates from S1, and the two heptad repeat regions form a fusion core, exposing a fusion peptide that fuses the viral and host membranes [74,75]. 

Understanding the structural components of MERS-CoV and functional proteins involved in the transmission of the virus is a key for the design of therapeutic agents. This design has been centered around developing targets towards the S protein (specifically towards the N-terminal RBD domain and other S1 regions) and towards HR1 and HR2 of the S2 subunit. 

### 4.1. IgG

#### 4.1.1. D12, F11, G2, and G4

The majority of the immunization strategies to develop coronavirus vaccines have focused on using a whole-inactivated virus, live-attenuated virus, recombinant protein subunit, or other genetic approaches, with particular emphasis on the S1 subunit [76,77]. 

A novel immunization strategy was developed using vaccine regimes involving full length of S1 subunit protein to induce a response of a broad range of neutralizing antibodies against various MERS-CoV strains in mice [73]. The DNA and protein vector constructs, based on the England1 strain spike, yielded a variety of antibodies named D12, F11, G2, and G4 [77]. Results showed that when these antibodies were tested in both mice and non-human primates, and a cross-neutralization activity across eight different MERS-CoV strains in vitro was measured. The most potent antibodies, D12 and F11, bound the RBD on opposite sides, indicating their potential for additive effects if used together. However, the antibody F11 was unable to neutralize the Bisha1 strain due to an aspartic acid to glycine substitution at residue 509. While D12 did not exhibit this resistance, crystallization of this antibody as a Fab in complex with the England1 RBD showed the importance of RBD residues W535 and E536 (both binding the glycan on the receptor DDP4). 

Interestingly, the antibodies G2 and G4 were unique in their property in that their neutralizing epitope was outside the RBD. Follow-up studies analyzing ex vivo selection for G2-escape variants revealed the importance of the residues S28F or G198D substitutions as critical for G2 binding to a region comprised of two loops atop S1-N-terminal domain (ST-NTD), preventing MERS-CoVs attachment to host the DPP4 receptor [78]. G4 epitope consisted of a glycosylated loop between the HR1 and HR2 of the S2 subunit. The antibody and loop interaction impeded the necessary conformational changes of the S2 subunit needed for membrane fusion after viral attachment to DPP4 [77,79]. 

It is important to recognize that while G2 and G4 may have high potency compared to other antibodies that target outside the RBD, individually, they may not be as effective as those targeting RBD or S2 region specifically. 

##### 4.1.2. m336, m337, and m338 

When faced with a novel human virus such as MERS-CoV, it is important to adopt an approach that can rapidly develop potential therapeutics against the target. As broad-spectrum antivirals have not proven effective in a clinical setting against MERS-CoV, a rapid antibody selection approach, targeting the virus from a large non-immune antibody library was used [80]. This approach used B cells from 40 healthy donors, and a novel IgM library consisting of around 10^11^ antibodies was generated. The three antibodies m336, m337, and m338 showed significantly high potent neutralizing activity to the recombinant MERS-CoV S protein RBD [80]. All three antibodies exhibited neutralization against the pseudovirus system (HIV luciferases virus pseudotyped with MERS-CoV S protein) and live MERS-CoV virus, with m336 being the most potent. Epitope mapping revealed that binding sites of the antibodies are within the RBD (overlapping) but at distinct epitopes, suggesting that the antibodies neutralize the virus by competing for the binding of the host receptor. When compared to the humanized antibody 4C2H, m336 showed a 100-fold higher potency. However, realizing that both antibodies bind at different sites on the RBD, a combination of two might prove to deliver a synergistic effect against wild type and escape mutants of MERS-CoV [81,82].

Acknowledging the lack of an appropriate animal model of MERS-CoV infection due to the difficulty of the virus to infect small animals (divergence in the DDP4 receptor sequences), a follow-up study to test the effectiveness of m336 made use of transgenic mouse models that were transduced with human DPP4 vectors to make them susceptible to MERS-CoV infection [83,84]. Treatment of mice both before or after challenge with m336 antibody protected mice in a dose-dependent fashion as determined by recovery from clinical manifestations and barely detectable levels of lung virus titers. However, when a rabbit animal model was used, only a prophylaxis treatment with m336 reduced viral RNA titers, as post-exposure treatment was not effective [85]. The differences in results could be due to differences observed in disease progression in rabbits compared. Therefore, further testing across various models would provide a more thorough understanding of the potential of m336 as a therapy [86,87]. 

##### 4.1.3. LCA60

In search of a rapid way to develop appropriate treatments for new and emerging zoonotic viruses, the antibody LCA60 was the first human neutralizing antibody derived from the memory B-cells of MERS-CoV patients. The antibody was isolated from the B-cell culture of extracted serum samples derived from a 49-year old man from Qatar who was hospitalized due to severe respiratory failure [88]. 

When compared to the non-neutralizing antibody LCA57, LCA60 was able to display neutralizing activity across London1/2012, EMC/2012, and Jorand-N3/2012 strains with high potency, which is comparable with the neutralizing profile of m336 and more potent than 3B11. Escape studies of monoclonal antibody resistant mutants showed that the mutations of V33A in the N-terminal and E536A highlighted the ability of LCA60s to act by targeting not only the RBD but other regions of the S protein. In contrast, further epitope mapping showed the importance of residues T489, K493, E565, and E536 within the RBD in binding to DPP4. 

Prophylactic and post-exposure of LCA60s in mice sensitive to MERS-CoV infection showed a significant reduction in lung viral titers, with undetectable levels noted by day 5 [88]. Further testing with stringent models showed similar results, with comparable results being reported when LCA60 is administered intranasally, highlighting the potential for a nebulization route of administration as an alternative to the parenteral route. 

The benefits of clinical treatments using convalescent sera across viral infections, including the Spanish flu, SARS-COV, and H1N1 influenza, have been documented. The human-derived antibody LCA60 is one such option, as preclinical studies have shown its potential in neutralization multiple isolates of MERS-CoV infections. However, even though its important binding residues are conserved in all-natural isolates to date, mutations of E536A and V33A within escape variants render the antibody ineffective. Additional challenges of isolation of human antibodies include lack of timely available convalescent sera or insignificant antibody titers that prevent adequate production. 

##### 4.1.4. MERS-GD27 and MERS-GD33

Further studies related to enhancing of the LCA60 antibody activity led to the generation of human monoclonal antibodies by cloning antibody genes from primary B-cells of infected patients to study the germline origins of their genes [89]. 

The recombinant S protein was used to screen B-cells from a MERS-CoV patient during the 2015 outbreak in Korea [90]. From the selected B-cells that were cloned, 13 specific human antibodies were identified. Among them, MERS-GD27 and MERS-GD33 were the most potent, as determined by their neutralization activities against MERS-CoV pseudovirus using the Huh cells and live MERS-CoV stock (hCoV-EMC). Epitope mapping of the two antibodies revealed distinct regions of S protein binding with additional crystal structural analysis. In the case of MERS-GD27, the interaction was mediated by the interaction with 16 residues from the β5 strand, β5-β6 loop, β6-β7 loop, β7 strand, and β7-β8 loop of the RBD, blocking its interaction with the DPP4 receptor [90]. While epitope and mutagenesis analyses were suggestive of different activities of the MERS-GD27 and MERS-GD33, more detailed work should map out their direct site of activity, particularly for MERS-GD33. Additional in vivo study with MERS-GD27 in a human DPP4 (hDPP4) transgenic mouse model showed its protective factors as its administration before and after a lethal challenge with MERS-CoV. This administration led to the decrease in clinical symptoms, lung viral titers, and overall higher survival rates in comparison to a control group receiving irrelevant monoclonal antibodies [89]. 

When compared with other antibodies that bound RBD of MERS-CoV, the m336 and MERS-GD27 antibodies appeared to have the largest epitope overlap and the lowest IC_50_ values for neutralization of the pseudovirus when compared to MERS-27, MCA1 (see below), and D12 [77,82,91,92].

Genetic analysis also showed a high diversity of germline origins of the antibodies from that patient, with a high frequency of the immunoglobulin heavy variable precursor *IGHV1-69* allele, which could highlight this gene as the common precursor for MERS-CoV antibodies. The *IGHV1-69* allele has also been recognized as being heavily involved in antibodies produced against other diseases, including HIV, influenza, and hepatitis [93,94,95]. This, along with the low levels of somatic hypermutations required for maturation, is suggestive that antibody production against MERS-CoV could occur at a faster rate in comparison to other diseases (e.g., HIV) that require great divergence from their germline origin along with a complex maturation process. 

##### 4.1.5. 4C2 and 2E6

The sudden emergence of MERS-CoV infection was followed by applying many rapid preventive treatment measures to counter the virus. With no promising vaccines or drugs in development, the identification of neutralizing antibodies that could be used against MERS-CoV infection was pursued [82].

While acknowledging the work done from identifying antibodies from antibody libraries, a conventional method of isolating antibodies from immunized animals was used [82]. From a panel of clones that reacted against recombinant MERS-RBD proteins in immunized mice, the clones 4C2 and 2E6 showed high potency in inhibiting the interaction between RBD and host receptor hCD26/DPP4. In further testing, both antibodies could inhibit the pseudotype and infectious MERS-CoV virus entry into the hepatocyte cell line Huh7. X-ray crystallography analysis showed that the 4C2 paratope used all six complementary determining regions (CDRs) to block virus attachment to hCD26. More detailed analysis showed that 4C2 mainly interacts with different amino acids than those of MERS-RBD, particularly residues Y397-N398, K400, L495-K496, P525, V527-S532, W535-E536, and D539-Q544, which lead to the conclusion that the inhibition of receptor binding by 4C2 is attributed to the steric hindrance between 4C2 and hCD26 that prevents viral attachment. 

To further test the application of 4C2, a humanized version of the antibody was generated, maintaining its neutralization ability to the same extent as the parental mouse antibody [82]. The humanized version of the antibody was further tested in mouse models. The result showed a significant decrease in virus titers in the lung by approximately two orders of magnitude when 4C2 was administered either in a pre- or post-challenge.

Even though the known RBD mutations across MERS-CoV variants do not involve the 4CS’s epitope, a combination of 2E6 and 4C2 may prove to be effective [82]. This will require further testing and characterization of 2E6, as initial attempts were not successful due to the inability to crystalize 2E6 with MERS-RBD. 

#### 4.2. ScFv Antibodies

##### 4.2.1. MERS-4 and MERS-27

The interaction between the RBD of MERS-CoV spike protein and its receptor DDP4 has certainly led to recognizing monoclonal antibody treatments directed against DPP4 in human cells [96,97]. However, with the understanding of DPP4’s important function in immunoregulation, antibodies directed against it might lead to various adverse effects in human use. Therefore, it is warranted to investigate treatments aimed at targeting the RBD specifically. 

A non-immune human single-chain variable fragment library display to isolate antibodies against the MERS-CoV RBD was used [91]. From the selected antibodies, MERS-4 and MERS-27 were the two that demonstrated high potency for neutralization of pseudovirus infections and clinical isolate hCOV-EMC from a MERS-CoV infected patient. Biochemical analysis of the two antibodies showed a mechanism of action involving the insertion of the antibody between the RBD and DPP4 receptor. Follow-up studies in epitope mapping have demonstrated that MERS-27 interacts with the C-terminal segment of the β6-β7 loop and β7 strand of the RBD of MERS-CoV, which overlaps with the DPP4 binding surface. On the other hand, MERS-4 binds with the β5-β6, β6-β7, and β7-β8 loops of the receptor-binding subdomain in RBD of MERS-CoV, which do not overlap with the DPP4 binding region [79,98,99]. These different sites of activity could explain why a synergistic effect was noted when combing the two antibodies. 

While synergistic effects were noted, this was only apparent at low concentrations because an increase in the concentrations of the two antibodies was only met with a modest degree of synergism. This was attributed to a possible steric hindrance; however, this would warrant further investigations as the antibodies have different activity sites. Additionally, the more potent antibody MERS-4 became much less potent upon specific spike protein mutations, such as D455A, E513A, and R542A. Further testing with animal models and future clinical trials could better understand the pharmacokinetics and effectiveness of the antibodies for future prophylactic use. 

##### 4.2.2. MCA1

The majority of in vivo studies rely on the use of murine models. A non-human primate model (marmoset) was used to investigate the activity of antibodies [100]. This model is much closer to mimicking the disease progression observed in humans, making it an opportunity to understand the pathogenesis and potential treatment strategies better.

MCA1 was isolated from a phage display library and converted into full-length human IgG1 for further testing [101]. In vitro testing showed a potent dose-dependent neutralization activity against MERS-CoV in Vero E6 cells. Crystallization analysis of the MCA1/MERS-CoV RBD complex showed direct contact of MCA1 with the RBD with all six CDRs with steric clashes being noted. The specific interacting residues of the MCA1 included F26, S27, and S28 in H1; G98, D99, T100, and R103 in H3; and G91 in L3. Additionally, MCA1 demonstrated both prophylactic and treatment efficacy when administered to common marmosets as clinical outcomes improved while viral titers significantly decreased. 

#### 4.3. Peptides

##### HR2L, HR2P, and HR2P-M2

While the majority of the dedicated research into antibody treatment against MERS-CoV have focused on their interaction with the S1 domain of the spike glycoprotein, another study focused on the S2 domain in the hopes of better understanding the fusion and entry mechanism that can lead to the development of appropriate inhibitors [100]. 

As in SARS-CoV, the S2 domain of the spike protein of MERS-CoV is involved in the fusion of virus into the target cell through the activation of S protein’s residues 987-1062 in HR1 and 1263-1279 in the HR2 domains that creates a stable six-bundle (6-HB) fusion core that brings the membranes of the cells closer together [73,75,100]. Two peptides were synthesized from the HR1 and HR2 domains, termed HR1P and HR2P, respectively, and were tested for their ability to inhibit viral entry and replication [75]. Only HR2P exhibited a potent inhibition in preventing effector cells expressing MERS-CoV S protein (293T/MERS/EGFP) to fuse to the target cell expressing the DPP4 receptor (Huh cells). Additionally, only HR2P could inhibit replication of MERS-CoV inside Vero E6 cells in a dose-dependent manner. This inhibition can be attributed to HR2P, specifically interacting with the viral HR1 domain, to form the heterogenous 6-HB that prevents viral fusion with the host cell [75]. The difference in activity levels between the HR1P and HR2P may be due to the length of the peptides, HR1P being a shorter peptide compared to HR2P, and, then, avoiding the formation of the typical fusion structure. In general, the length of the peptide synthesized had an improving effect on inhibition. Furthermore, the addition of two-point mutations into HR2P-M1 and a seven-point mutation into HR2P-M2 peptide led to stronger salt bridges that increased the stability, solubility, and anti-MERS-CoV activity of the peptides.

Following the results of this study, the authors proposed the development of HR2P and HR2P-M2 analogs. It was expected that HR2P-M2 would show potent inhibition of pseudoviral cell mediation fusion along with various MERS-CoV isolates that had Q1020H or Q1020R mutations in their S protein HR1 domain [102]. Since mice are naturally not prone to MERS-CoV infection, transgenic mice containing the Ad5-hDPP4 were used to test HR2P-M2 potency in vivo [102]. Results of an intranasal administration of HR2P-M2 before and after challenges with MERS-CoV EMC/2012 led to reduced viral titers in the lungs, with even greater potency being noted when the analog was combined with IFN-β. In summary, the results reflect the potential of HR2P-M2 as a prophylactic or therapeutic treatment for MERS-CoV infected patients at the early stage of the infection to prevent viral entry, similar to anti-HIV peptide T20 (enfuvirtide) that is used for HIV infections [103].

## 5. Conclusions

The burden placed by SARS-CoV-2 on the health, societal, and economic sectors continues to increase globally and will do so until appropriate therapeutic approaches can be implemented. The lessons learned from studies using neutralizing antibodies in similar diseases of the past, including SARS and MERS, can serve as a foundation and guideline in designing antibodies specific to SARS-CoV-2.
